# A Polyvinylpyrrolidone-Based Supersaturable Self-Emulsifying Drug Delivery System for Enhanced Dissolution of Cyclosporine A

**DOI:** 10.3390/polym9040124

**Published:** 2017-03-27

**Authors:** Dae Ro Lee, Myoung Jin Ho, Young Wook Choi, Myung Joo Kang

**Affiliations:** 1 College of Pharmacy, Dankook University, 119 Dandae-ro, Dongnam-gu, Cheonan, Chungnam 330-714, Korea; pharmdr7@naver.com (D.R.L.); butable@gmail.com (M.J.H.); 2 College of Pharmacy, Chung-Ang University, 221 Heuksuk-dong, Dongjak-gu, Seoul 156-756, Korea; ywchoi@cau.ac.kr

**Keywords:** cyclosporine A, self-emulsifying drug delivery system (SEDDS), supersaturation, apparent concentration, precipitation inhibitor, polyvinylpyrrolidone

## Abstract

A novel supersaturable self-emulsifying drug delivery system (S-SEDDS) of cyclosporine A (CyA)—a poorly water-soluble immunosuppressant—was constructed in order to attain an apparent concentration–time profile comparable to that of conventional SEDDS with reduced use of oil, surfactant, and cosolvent. Several hydrophilic polymers, including polyvinylpyrrolidone (PVP), were employed as precipitation inhibitors in the conventional SEDDS, which consists of corn oil-mono-di-triglycerides, polyoxyl 40 hydrogenated castor oil, ethanol, and propylene glycol. PVP-incorporated pre-concentrate (CyA:vehicle ingredients:PVP = 1:4.5:0.3 *w/v/w*) spontaneously formed spherical droplets less than 120 nm within 7 min of being diluted with water. In an in vitro dialysis test in a biorelevant medium such as simulated fed and/or fasted state intestinal and/or gastric fluids, PVP-based S-SEDDS exhibited a higher apparent drug concentration profile compared to cellulose derivative-incorporated S-SEDDS, even displaying an equivalent concentration profile with that of conventional SEDDS prepared with two times more vehicle (CyA:vehicle ingredients = 1:9 *w/v*). The supersaturable formulation was physicochemically stable under an accelerated condition (40 °C/75% RH) over 6 months. Therefore, the novel formulation is expected to be a substitute for conventional SEDDS, offering a supersaturated state of the poorly water-soluble calcinurin inhibitor with a reduced use of vehicle ingredients.

## 1. Introduction

Cyclosporine A (CyA) is a potent cyclic polypeptide which has been prescribed to prevent organ rejection in cases of allogeneic liver, heart, and kidney transplantation [1,2]. The bioavailability (BA) of the calcinurin inhibitor following oral administration is low and variable, owing to its low solubility (6.6 μg/mL in water at 37 °C) in the gastrointestinal (GI) tract and pre-systemic metabolism [3,4]. To increase intestinal absorption while lessening the pharmacokinetic variations of the immunosuppressant, the manufacturer formulated a self-emulsifying drug delivery system (SEDDS), which mainly consists of oil (corn oil-mono-di-triglycerides), surfactant (polyoxyl 40 hydrogenated castor oil), and cosolvent (ethanol and propylene glycol) [5]. The pre-concentrate forms oil-in-water droplets upon contact with gastric fluids, placing the hydrophobic compound in a fine droplet in an aqueous environment, with no precipitation. This leads to a more profound dissolution profile, providing a large interfacial area for drug diffusion [6,7]. The marketed product (Sandimmune Neoral^®^, Novartis Pharma, Basel, Switzerland) provides more consistent and predictable pharmacokinetic behavior regardless of food intake, decreasing both inter- and intra-patient variability in drug concentrations in the blood [8,9]. However, the SEDDS-based marketed product contains considerable quantities of vehicle ingredients, in particular polyoxyl 40 hydrogenated castor oil (Kolliphor RH40), which could cause severe GI side effects, nephrotoxic and anaphylactoid reactions after long-term oral administration [10]. Kiss et al. (2013) even reported that an amount of surfactant corresponding to that given with clinical CyA doses caused remarkable cytotoxicity to endothelial and epithelial cells [11]. In this regard, the use of vehicle ingredients—especially Kolliphor RH40—should be minimized so that intake levels are tolerable.

Over the last decade, a supersaturation technique has been demonstrated to be an effective approach to increasing the aqueous solubility and intestinal absorption of poorly water-soluble compounds [12]. In supersaturable formulations, drug concentration temporarily exceeds equilibrium solubility when exposed to gastrointestinal fluids. Precipitation inhibitors such as polymers, surfactants, and cyclodextrins inhibit and/or slow drug precipitation in an aqueous medium by interfering with drug crystal nucleation and/or growth via interactions with drug compounds or by changing the properties of the medium [13,14,15]. Several previous studies reported that hydrophilic polymers such as hydroxypropyl methylcellulose (HPMC), hydroxypropyl cellulose (HPC), and polyvinylpyrrolidone (PVP) prolonged the supersaturated state in an aqueous medium by forming hydrogen bonds between drug molecules and/or by adsorbing on drug crystals and subsequently sterically hindering the drug crystallization process [16,17,18,19]. Given these findings, we assumed that employing the supersaturation technique in a conventional self-emulsifying system could effectively retard precipitation of the poorly water-soluble immunosuppressant in aqueous medium and reduce the use of vehicle components compared to conventional SEDDS. Previous studies with poorly water-soluble drugs such as paclitaxel [20], PNU-91325 [21], and AMG 517 [22] support the notion that supersaturable SEDDS (named S-SEDDS) offer apparent concentration–time profiles and oral absorption higher than and/or comparable to conventional SEDDS, with reduced use of oils and surfactants.

The aim of the present study was to build a novel S-SEDDS for CyA to provide an in vitro apparent concentration profile comparable to that of conventional SEDDS, with the reduced use of oil, surfactant, and co-surfactant. Supersaturable CyA systems were prepared by incorporating polymeric materials such as HPC, vinylpyrrolidone–vinyl acetate copolymers (Kollidon VA64), and PVP into conventional SEDDS as precipitation inhibitors. The physical characteristics of the system were assessed by morphology, self-emulsification time, droplet size, and surface charge. Moreover, the in vitro concentration–time profiles of CyA from the supersaturable systems were evaluated using four different types of biorelevant media: fasted state simulated gastric fluid (FaSSGF), fed state simulated gastric fluid (FeSSGF), fasted state simulated intestinal fluid (FaSSIF), and fed state simulated intestinal fluid (FeSSIF), and were then compared with those of conventional SEDDS.

## 2. Materials and Methods

### 2.1. Materials

Drug powder (purity >98.0 wt %) was kindly supplied by Chong Kun Dang Pharm (Seoul, Korea). Kolliphor RH40, kollidon VA64, and PVP K17 were kindly provided by BASF (Ludwigshafen, Germany). Corn oil-mono-di-triglycerides (Maisine 35-1) was obtained from Gattefosse (Saint Priest, France). Low-substituted HPC was obtained from Shin-Etsu Chemical Co. (Tokyo, Japan). Ethanol and propylene glycol were purchased from Duksan Pure Chemicals (Seoul, Korea). Acetonitrile and methanol were acquired from J.T. Baker (Phillipsburg, NJ, USA). All other chemicals were of reagent and/or analytical grade and were used without further purification.

### 2.2. Preparation of CyA-Loaded Self-Emulsifying Formulations

The compositions of the tested SEDDS and S-SEDDS formulations are presented in Table 1 and were prepared in five times greater volume than currently necessary for further experimentation. F1 was formulated to reflect the composition of the marketed product, based on previous literature [23]. Conventional SEDDS formulations (F1, F2, and F3) were prepared by dissolving the drug in an isotropic mixture of Maisine 35-1, Kolliphor RH40, ethanol, and propylene glycol at 40 °C, using a magnetic stirrer. S-SEDDS formulations (F4–F9) were prepared by adding polymeric materials to the SEDDS formulation (F3) and then vortexing vigorously to obtain a uniform solution and/or suspension. All formulations were stored at room temperature before further experiments.

### 2.3. Morphology of CyA-Loaded Self-Emulsifying Formulations

The appearance of CyA-loaded formulas after dilution with distilled water was examined using a transmission electron microscope (TEM). Each pre-concentrate (100 μL) was added to distilled water (1 mL) and gently vortexed for 5 min. A drop of sample was placed on a film-coated copper grid and dipped into uranyl acetate solution for 10 s to strain the oil droplets. Afterward, the dried sample was observed by TEM (JEM 1010, JEOL, Tokyo, Japan) at an acceleration voltage of 80 kV.

### 2.4. Droplet Size and Zeta-Potential

After dilution of CyA-loaded pre-concentrate (100 μL) with distilled water (10 mL), an appropriate volume of sample was loaded into a cuvette. Each cuvette was placed in a thermostated sample chamber at 25 °C. Then, the mean globular size, its homogeneity, and the zeta-potential of each aliquot were estimated by dynamic light scattering (DLS) using a Zetasizer Nano ZS equipment (Malvern Instruments, Malvern, UK). The wavelength used was 635 nm, and light scattering was monitored at 90°. All measurements were carried out in triplicate.

### 2.5. Self-Emulsification Time

Each formulation containing 100 mg of CyA was added dropwise into 500 mL of simulated gastric fluid in a glass beaker and gently stirred at 50 rpm with a magnetic stirrer. The time required to form a homogenous solution with no agglomerates and/or lumps was visually assessed. 

### 2.6. In Vitro Dialysis Test

The in vitro apparent concentration profiles of CyA from the self-emulsifying formulations were assessed according to the USP XXVIII paddle method. Each preparation containing 100 mg of CyA was transferred into a vessel containing 500 mL of biorelevant medium (i.e., FaSSGF, FeSSGF, FaSSIF, or FeSSIF). The compositions and the procedures used to prepare the biorelevant media are the same as those described previously [24,25,26,27,28]. Briefly, FaSSGF was prepared with 0.08 mM of sodium taurocholate, 0.02 mM of lecithin, 45.2 mM of sodium chloride, and hydrochloric acid in 1000 mL of distilled water (pH 1.6, osmolality 120 ± 3 mOsm∙kg^−1^). FeSSGF was prepared with 237.0 mM of sodium chloride, 17.1 mM of acetic acid, 29.7 mM of sodium acetate, milk/acetate buffer (1:1), and hydrochloric acid in 1000 mL of distilled water (pH 5.0, osmolality 400 ± 10 mOsm∙kg^−1^). FaSSIF was prepared with 3.0 mM of sodium taurocholate, 0.2 mM of lecithin, 19.1 mM of maleic acid, 68.7 mM of sodium chloride, and 34.8 mM of sodium hydroxide in 1000 mL of distilled water (pH 6.5, osmolality 180 ± 10 mOsm∙kg^−1^). FeSSIF was prepared with 10.0 mM of sodium taurocholate, 2.0 mM of lecithin, 5.0 mM of glyceryl monooleate, 0.8 mM of sodium oleate, 55.0 mM of maleic acid, 125.5 mM of sodium chloride, and 81.7 mM of sodium hydroxide in 1000 mL of distilled water (pH 5.8, osmolality 390 ± 10 mOsm∙kg^−1^). The temperature in the vessels was maintained at 37 ± 0.5 °C, and the paddle revolution speed was 50 rpm. Approximately 1 mL aliquots were withdrawn at 0.16, 0.33, 0.5, 1.0, 2.0, 4.0 and 6.0 h, and were centrifuged at 13,000 rpm for 5 min. After sampling, an equal volume of fresh aqueous media at the same temperature was added to the vessel to keep the volume of media constant. After centrifugation, the supernatant was collected and then appropriately diluted with methanol to analyze the drug concentration using HPLC, as described below.

### 2.7. HPLC Assay for CyA

The concentration of CyA in the samples was quantitatively analyzed using an isocratic HPLC system. The Waters HPLC system consisted of a pump (Model 515 pump), an autosampler (Model 71P auto sampler), and a UV detector (Model 486 UV detector) equipped with a Luna C8 column (4.6 mm × 150 mm, 3 μm). The mobile phase consisted of a mixture of acetonitrile, distilled water, and phosphoric acid (750:250:1, *v/v/v*) at a flow rate of 1.2 mL/min. The column temperature was set at 60 °C in a column oven and the eluent was monitored at 220 nm. The calibration curve was linear in the drug concentration range of 5–100 μg/mL (*y* = 36,221*x* + 4771, *r*^2^ = 0.9999). Intra-day and inter-day precision ranged from 0.24% to 0.82%, and from 2.57% to 3.31%, respectively. 

### 2.8. Stability Test

Approximately 10 g of CyA-loaded S-SEDDS (F8) was placed into a glass scintillation vial and stored in a stability chamber maintained at 40 ± 2 °C/75 ± 5 RH % for 6 months. Samples were then analyzed for droplet size after dilution with distilled water, self-emulsification time, and in vitro concentration–time profile in FeSSIF medium.

## 3. Results and Discussion

### 3.1. Morphological and Physical Characteristics of Self-Emulsifying Formulations

**Figure 1:**
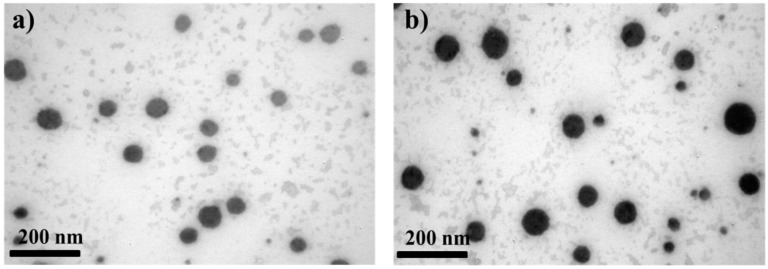
TEM micrographs of (**a**) CyA-loaded conventional SEDDS (F1) and (**b**) PVP-incorporated supersaturable SEDDS (S-SEDDS, F9).

Different conventional (F1–F3) and supersaturable (F4–F9) self-emulsifying formulas were prepared, and their physical properties were characterized in terms of morphology, globular size, zeta-potential, and self-emulsification time. TEM observation revealed that all SEDDS and S-SEDDS compositions were comprised of spherical globules in the size range of 20 to 100 nm (Figure 1), with no aggregation and/or precipitation. There was no morphological difference between conventional and supersaturable systems visible upon microscopic observation, indicating that the vehicle ingredients effectively formed drug-loaded fine emulsions, regardless of the type of hydrophilic polymer incorporated.

The droplet size of the SEDDS and S-SEDDS formulations after dilution with distilled water was determined using the DLS method. Globular size is one of the crucial factors that governs the rate and extent of drug release from the droplets as well as their in vivo colloidal stability after oral administration [29,30]. The droplet size of formulation F1 was approximately 40 nm (Table 1), which is quite in agreement with that of the marketed product, as previously reported (26.2 nm) [31]. The droplet size of F3—which contained only half as much vehicle as did F1—was increased to 102 nm, as the increase in the drug-to-vehicle ratio resulted in a gradual increase in the droplet size of the emulsion. On the other hand, the droplet size of the cellulose derivative—(F4) or PVP derivatives-incorporated S-SEDDS (F5–F9) was comparable to that of conventional SEDDS (F3) (between 100 nm and 123 nm), indicating that the hydrophilic precipitation inhibitor did not adsorb and/or incorporate onto the surface of the droplets. The low polydispersity index (PDI) of <0.4 of all SEDDS and S-SEDDS formulations indicated a monodisperse size distribution. The surface charge of all formulations was neutral or slightly negative, probably because of the free fatty acids in the oily ingredient.

The emulsification efficiency of the SEDDS and S-SEDDS formulations was estimated by determining the time taken to form a clear dispersion upon dilution with water. Preferably, self-emulsifying systems should rapidly disperse in an aqueous medium under gentle agitation. Conventional SEDDS (F1–F3) were easily dispersed in aqueous medium within 8.5 min (Table 1), forming a microemulsion with a bluish reflection. The good emulsifying capability of Kolliphor RH40 might accelerate the rate of dispersal of the pre-concentrates in the aqueous medium. Moreover, ethanol and propylene glycol cosurfactants may have aided the emulsification behavior of the surfactant by lowering the interfacial tension during the dispersal and/or formation of the oil droplet. On the other hand, when the cellulose derivative was employed as precipitation inhibitor in the formulas (F4), the emulsification time increased drastically to 15.5 min. The incorporation of the cellulose derivatives might cause an increase in the viscosity of the pre-concentrates, which probably delayed the spreading of the formulas in the aqueous medium. On the other hand, the addition of a small amount of vinyl polymers to the pre-concentrate (F5–F9) did not alter the emulsification time of the pre-concentrate (F3), with emulsification times between 5.4 and 6.5 min. Thus, S-SEDDS formulas containing Kollidon VA64 or PVP as precipitation inhibitors were expected to be promptly emulsified upon contact with the aqueous medium, providing high drug concentrations in the initial period.

### 3.2. In Vitro Dialysis Test

The influence of formulation variables such as the proportion of the drug to the SEDDS vehicle and the type and amount of precipitation inhibitor on the apparent concentration–time profile of CyA was assessed in biorelevant media (FaSSGF, FeSSGF, FaSSIF, and FeSSIF). Although co-administration of the marketed product with food caused a small (<20%) decrease in oral BA, the self-emulsifying system-based marketed product significantly reduced the effect of food on intestinal absorption and caused an increase in dose linearity compared to the crude emulsion (Sandimmune^®^) [8,9]. Thus, in order to replace the marketed product with a high pay-loaded supersaturable system, the latter should provide a comparable concentration profile with that of the marketed product in both fasted and fed simulated fluids.

**Figure 2:**
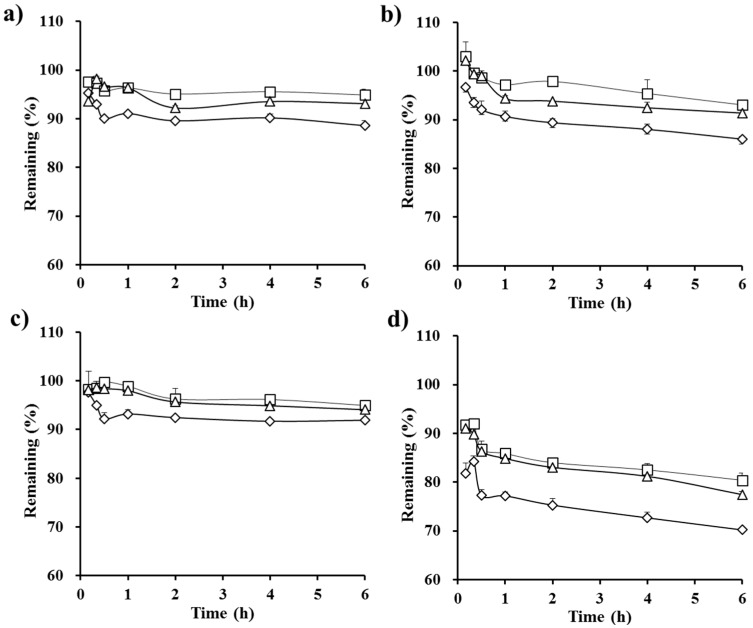
In vitro apparent concentration profiles of CyA from the conventional SEDDS formulations in (**a**) fasted state simulated gastric fluid (FaSSGF); (**b**) fed state simulated gastric fluid (FeSSGF); (**c**) fasted state simulated intestinal fluid (FaSSIF); and (**d**) fed state simulated intestinal fluid (FeSSIF) medium prepared with different amounts of vehicle components: 900 mg (F1, □), 675 mg (F2, **△**), and 450 mg (F3, ◊). The amount of drug in each formulation was equivalent to 100 mg, and the data are expressed as mean ± SD (*n* = 3).

First, the effect of the amount of vehicle in conventional self-emulsifying formulations containing an equal amount of CyA (100 mg, F1–F3) on the drug apparent concentration profile was evaluated (Figure 2). The apparent drug concentration in all types of biorelevant media reached a plateau within 30 min in all SEDDS formulations tested (F1–F3), indicating that these pre-concentrates promptly formed drug-loaded fine droplets upon dilution with aqueous medium, regardless of the quantity of vehicle. In FaSSGF, FeSSGF, and FaSSIF media, the drug concentration from F1 and F2 was over 90% for an extended period. However, in the case of F3 (which contained only half the amount of vehicle as F1), the amount of drug remaining in those media as a function of time were slightly lower (<7%) than those of F1. The reduction in the amount of vehicle ingredients lowered the drug solubilization capacity, thus accelerating drug precipitation after dilution in the aqueous medium. On the other hand, the influence of formulation variables on the concentration profile of CyA was more noticeable in FeSSIF medium, because the colloidal stability of the drug-loaded droplets in medium with a significant amount of the lipid and surfactant materials is generally much lower than in other media. After reaching a plateau within 20 min in all formulations, the drug remaining (%) from the conventional formulations gradually decreased as time elapsed; the drug remaining (%) from F1, F2, and F3 was 81%, 77%, and 70%, respectively, at 6 h. In the next study, different hydrophilic polymers were incorporated into the SEDDS F3 formula, and the apparent concentration profiles were primary assessed in FeSSIF medium in order to better understand the influence of the polymers on the drug concentration profile.

**Figure 3:**
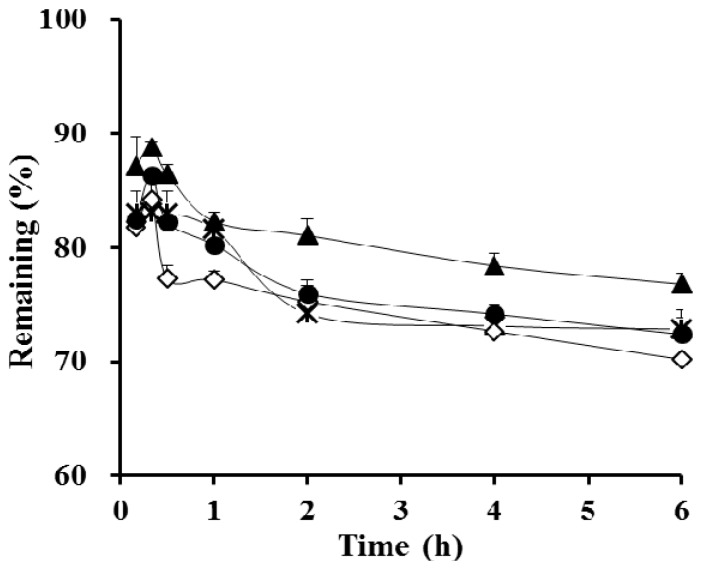
In vitro apparent concentration profiles of CyA in FeSSIF medium from the conventional SEDDS formulation (F3, ◊) and from S-SEDDS formulations containing different polymeric materials as precipitation inhibitors: HPC—(F4, **×**), Kollidon VA64—(F5, ●), and PVP-incorporated S-SEDDS (F6, ▲) formulas. The amount of polymeric materials in the S-SEDDS formulations was fixed at 15 mg, and the data are expressed as mean ± SD (*n* = 3).

Different kinds of polymeric materials (HPC, Kollidon VA64, and PVP) were incorporated into the SEDDS F3 formula as precipitation inhibitors to obtain a similar concentration–time profile as that of F1, while using only half the amount of vehicle contained in F1. F4 was an opaque suspension, as the cellulose derivative was not readily soluble in the oily vehicle. F5 and F6 were transparent, as the polymers were dissolved in the SEDDS vehicle. The apparent drug concentration–time profiles of the S-SEDDS formulations are depicted in Figure 3. In cellulose derivative-incorporated S-SEDDS (F4), there was no noticeable increase in apparent drug concentration in the aqueous medium after 1 h. Although HPC was reported to be an effective precipitation inhibitor of CyA in solid dispersion [32], the polymer suspended in the oily composition could not quickly access free drug molecules and/or crystals liberated from the droplets. In contrast, the incorporation of PVP into conventional SEDDS (F6) significantly increased the apparent drug concentration during the experimental period (Figure 3). In the case of F6, after reaching a maximum level within 20 min, the calcinurin inhibitor concentration in FeSSIF medium was maintained over 75% for 6 h. This observation is consistent with a previous report that a supersaturable SEDDS containing 0.5% PVP polymer was able to slow precipitation and maintain a high indirubin concentration for 2 h in an aqueous medium upon dilution [33]. Zhang et al. (2011) also reported that the addition of PVP polymer effectively prolonged the supersaturated state by retarding precipitation kinetics [16]. Hydrogen bonding between the PVP polymer with a carbonyl group as a hydrogen bond acceptor and the drug molecule—a polypeptide containing both hydrogen bond donors and acceptors—might effectively slow drug nucleation and/or crystal growth. Balani et al. (2010) reported that PVP has a higher propensity to form hydrogen bonds with functional groups exposed at the crystal surface [34]. 

**Figure 4:**
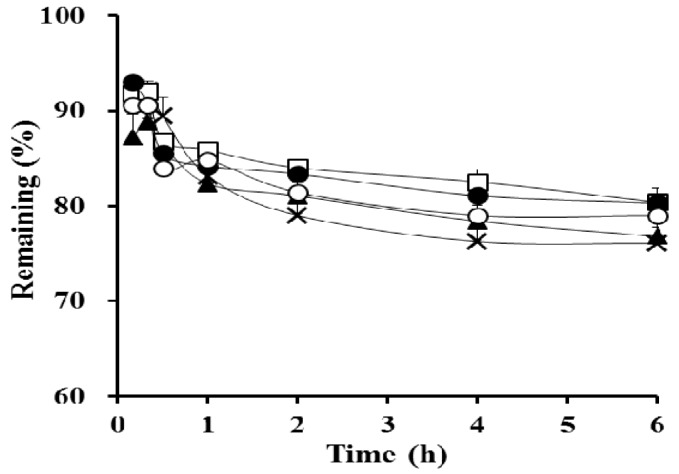
In vitro apparent concentration profiles of CyA in FeSSIF medium from the conventional SEDDS formulation (F1, □) and from PVP-based S-SEDDS formulations prepared with different amounts of hydrophilic polymer: 15 mg (F6, ▲), 20 mg (F7, **×**), 30 mg (F8, ●), and 40 mg (F9, ○). Data are expressed as mean ± SD (*n* = 3).

**Figure 5:**
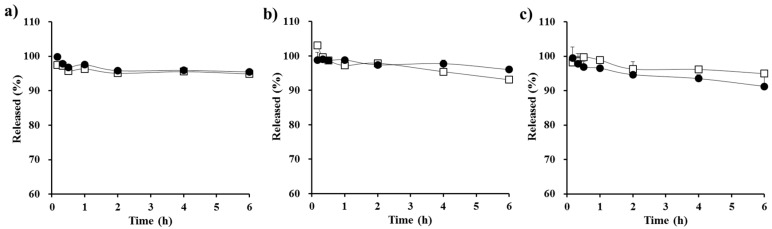
In vitro apparent concentration profiles of CyA from the conventional SEDDS (F1, □) and from the optimized PVP-based S-SEDDS formulation (F8, ●) in different biorelevant media; (**a**) FaSSGF; (**b**) FeSSGF; and (**c**) FaSSIF. Data are expressed as mean ± SD (*n* = 3).

A series of S-SEDDS formulations containing different amounts of PVP polymers (15, 20, 30, and 40 mg; F6–F9) were prepared, and their apparent concentration profiles were assessed in FeSSIF medium. The optimal amount of precipitation inhibitor in the supersaturable systems was previously reported to be drug- and formulation-specific [35]. As shown in Figure 4, as the proportion of the precipitation inhibitor increased, the drug concentration at 6 h was gradually increased. The concentration profile plateaued with 30 mg of the hydrophilic polymer (F8), providing a greater than 80% remaining at 6 h. On the other hand, the concentration profile of CyA from the F8 formula was equivalent to that of F1 not only in FeSSIF, but also in FaSSGF, FeSSGF, and FaSSIF (Figure 5), exhibiting a >91% remaining at 6 h. Taken together, this data shows that S-SEDDS F8—which had a comparable concentration–time profile to that of F1 in all biorelevant media—was selected as the optimal S-SEDDS composition for CyA.

### 3.3. Stability Test

The physicochemical stability of the optimized S-SEDDS formulation prepared with PVP polymer (F8) was evaluated by assessing droplet size after dilution, self-emulsification time, and concentration–time profile after storage under accelerated conditions (40 °C, 75 RH %). The droplet size and self-emulsification time of F8 with water were not markedly changed (*p* > 0.05) after storage under the accelerated conditions for 6 months (Table 2). Moreover, the apparent concentration profile of CyA from the stored supersaturable formulation was the same as that of F7 prior to storage (*p* > 0.05), providing an over 80% remaining in FeSSIF medium at 6 h (Table 2). Thus, we concluded that the S-SEDDS formulation of CyA was physicochemically stable.

## 4. Conclusions

To provide a high concentration–time profile of CyA with reduced use of vehicle, a novel S-SEDDS system was designed by adding PVP polymer (30 mg) as a precipitation inhibitor to a conventional SEDDS system. The optimized CyA-loaded S-SEDDS rapidly (<7 min) formed 114 nm-sized spherical droplets upon contact with aqueous medium. In an in vitro dialysis test in biorelevant medium, PVP polymer effectively slowed drug precipitation, providing a comparable concentration–time profile with the conventional SEDDS prepared with two times more oil, surfactant, and co-solvent. Moreover, the novel supersaturable system was stable, with no changes in droplet size, emulsification behavior, or concentration–time profile after 6 months of storage under accelerated conditions. From these findings, we conclude that S-SEDDS is beneficial for enhancing the apparent concentration profile and oral absorption of the poorly water-soluble immunosuppressant, with reduced use of oil and surfactant.

## Figures and Tables

**Table 1:** The compositions and physical characteristics of Cyclosporine A (CyA)-loaded self-emulsifying formulations.

Formulations	F1	F2	F3	F4	F5	F6	F7	F8	F9
*Compositions*									
CyA (mg)	100	100	100	100	100	100	100	100	100
Maisine 35-1 (μL)	320	240	160	160	160	160	160	160	160
Kolliphor RH40 (μL)	380	285	190	190	190	190	190	190	190
Ethanol (μL)	100	75	50	50	50	50	50	50	50
Propylene glycol (μL)	100	75	50	50	50	50	50	50	50
HPC (mg)				15					
Kollidone VA64					15				
PVP (mg)						15	20	30	40
*Physical characteristics*									
Size (nm)	39.4	36.7	102.5 *	123.0 *	120.6 *	113.4 *	104.2 *	114.1 *	120.1 *
Polydispersity index	0.33	0.26	0.28	0.28	0.29	0.32	0.35	0.38	0.25
Zeta potential (mV)	−5.1	−4.1	−5.4	−5.5	−3.8	−4.2	−5.6	−3.8	−4.4
Emulsification time (min)	8.5	7.4	6.2 *	15.5 *	6.1 *	6.2 *	5.4 *	6.5 *	5.8 *

Data on physical characteristics are expressed as mean values (*n* = 3). All standard deviations were <10% of the mean. * *p* < 0.05 versus the conventional self-emulsifying drug delivery system (SEDDS) F1. HPC: hydroxypropyl cellulose; PVP: polyvinylpyrrolidone.

**Table 2:** Physicochemical stability of the optimized S-SEDDS (F9) under accelerated storage conditions (40 °C/75% RH).

Parameters	Immediate after Preparation	After 6 Months Storage
Droplet size (nm) ^a^	114.1	126.3
Zeta potential (mV) ^a^	−4.2	−4.5
Emulsification time (min) ^a^	6.5	5.8
Remaining at 10 min (%) ^b^	93.0 ± 0.5	91.8 ± 1.5
Remaining at 6 h (%) ^b^	80.3 ± 1.5	81.4 ± 5.1

^a^ Data on physical characteristics are expressed as mean values (*n* = 3). All standard deviations were <10% of the mean values; ^b^ Data are expressed as mean ± SD (*n* = 3).
